# Atherosclerosis is associated with amyloid and tau pathology via blood–brain barrier dysfunction in the hippocampus of aged human brains

**DOI:** 10.1371/journal.pone.0324652

**Published:** 2025-06-11

**Authors:** Zhongman Jin, Nian Liu, Hui Wei

**Affiliations:** 1 State Key Laboratory of Common Mechanism Research for Major Disease, Institute of Basic Medical Sciences Chinese Academy of Medical Sciences, School of Basic Medicine Peking Union Medical College, Beijing, China; 2 Neuroscience Center, Chinese Academy of Medical Sciences, Beijing, China; The University of Texas Health Science Center at Houston, UNITED STATES OF AMERICA

## Abstract

Atherosclerosis, a chronic vascular condition characterized by lipid accumulation and arterial plaque formation, has emerged as a significant contributor to neurodegenerative diseases, including Alzheimer’s disease (AD). This study investigated the association between severe atherosclerosis and hippocampal changes in aged human brains, focusing on blood**–**brain barrier (BBB) dysfunction and its potential role in amyloid**-**beta (Aβ) and phosphorylated tau (pTau) pathology. Using multiplex immunohistochemical staining of postmortem brain tissue, we demonstrated that atherosclerosis-associated vascular damage leads to endothelial and smooth muscle cell apoptosis, exacerbates cerebral amyloid angiopathy (CAA), and promotes perivascular tau accumulation. These findings highlight a potential association between vascular health and neurodegeneration, offering insights into potential therapeutic targets for mitigating AD progression.

## 1. Introduction

Atherosclerosis is a chronic, progressive condition characterized by lipid accumulation, inflammation, and fibrous tissue deposition in arterial walls, leading to plaque formation and arterial stiffening [1,2]. While widely recognized as a major risk factor for cardiovascular diseases such as coronary artery disease and stroke [3], a growing body of evidence implicates atherosclerosis in the pathogenesis of neurodegenerative diseases, particularly Alzheimer’s disease (AD) [4–7].

Cerebrovascular dysfunction is a hallmark of atherosclerosis and manifests as impaired blood‒brain barrier (BBB) integrity, reduced cerebral blood flow, and vascular remodeling [5,8]. These vascular alterations compromise the brain’s homeostasis, setting the stage for neuronal injury and cognitive decline [9–11]. The hippocampus, a region critical for memory and learning, is especially vulnerable to both vascular and neurodegenerative insults [12]. In AD, the hippocampus exhibits hallmark pathological changes, including the formation of amyloid-beta (Aβ) plaques and phosphorylated tau (pTau) tangles, which disrupt neuronal networks and contribute to cognitive impairment [13]. Emerging studies suggest that vascular damage in atherosclerosis may exacerbate these pathologies, particularly through mechanisms such as endothelial and smooth muscle cell apoptosis, amyloid deposition in blood vessels, and localized tau aggregation [4,14,15].

Despite these findings, the relationship between severe atherosclerosis and the co-occurrence of vascular and neurodegenerative changes in the hippocampus remains incompletely understood. Most previous studies have focused on neuronal pathology without fully exploring the contributions of vascular dysfunction [16]. This study aims to address this gap by investigating the interplay between atherosclerosis-induced vascular degeneration and hippocampal neurodegeneration. We hypothesize that severe atherosclerosis (sAS) is associated with BBB dysfunction, vascular cell apoptosis, and amyloid and tau pathologies in the hippocampus, highlighting the potential interdependence of vascular and neuronal damage in AD.

## 2. Materials and methods

### 2.1. Specimen collection

In this study, 12 paraffin-embedded hippocampus–entorhinal cortex (HP–EC) tissue samples obtained from the National Human Brain Bank for Developmental and Functional Research (NHBBDF) at the Institute of Basic Medical Sciences on 15/3/2023, Chinese Academy of Medical Sciences were utilized. Ethical approval for the study was granted by the Institutional Review Board of the same institution, and informed consents (written) were obtained from the donors, ensuring that the use of these brain samples adhered strictly to National Human Brain Bank for Development and Function guidelines and the Declaration of Helsinki (approval number: 2022125). The severity of atherosclerosis in the brain tissues was scored by an expert neuropathologist on the basis of histopathological assessments of arterial plaques in the circle of Willis. Scores ranged from 0 (no atherosclerosis) to 3 (severe atherosclerosis); brain tissues given a score of 3 were included in the sAS group, while those given a score of 0 were included in the no atherosclerosis (nAS) group. The demographics of the subjects, including age and sex, are detailed in S1 Table.

### 2.2. Multiplex immunohistochemistry

A 5-marker multiplex immunohistochemistry panel for the detection of amyloid-beta (Aβ), phosphorylated tau (pTau), endothelial cells (CD31), smooth muscle cells (αSMA), and apoptotic cells (BAX) was developed to assess key pathological features. For multiplex immunofluorescence staining, we employed the TSA-based method (AlphaTSA^®^ Multiplex IHC Kit; Alpha X Biotechnology, China, Cat#: AXT37100031). Paraffin-embedded sections were dewaxed, rehydrated, and subjected to antigen retrieval in sodium citrate buffer (pH 6.0) at 95°C for 10 minutes. Hydrogen peroxide and blocking solution were used to reduce nonspecific binding. The sections were incubated with primary antibodies overnight at 4°C. The primary antibodies used were: mouse monoclonal anti-amyloid beta (1:2000, BioLegend, Cat#: 803001), mouse monoclonal anti-p-Tau (Ser202/Thr205, 1:2000, Invitrogen, Cat#: MN1020), mouse monoclonal anti-CD31 (1:1000, Proteintech Group, Cat#: 66065-2-Ig), rabbit polyclonal anti-alpha smooth muscle Actin (1:2000, Abcam, Cat#: ab5694), and rabbit monoclonal anti-BAX (D2E11, 1:500, Cell Signaling Technology, Cat#: 5023T). Followed by incubation with secondary antibodies for 10 minutes at 37°C. Each marker was visualized sequentially using fluorescent dyes (The corresponding relationship between markers and fluorescent dyes was as follows: Aβ‒XTSA480, pTau‒XTSA620, CD31‒XTSA570, αSMA‒XTSA780, and BAX‒XTSA520), and antigen stripping was performed between rounds of staining. DAPI was used to counterstain the nuclei. The entire tissue area on each slide was imaged using Axioscan7 automated slide scanning system (ZEISS, Germany) with a 20**×** objective lens. The fluorescence channel corresponded to the exposure time as follows: DAPI‒1 ms, XTSA480‒3 ms, XTSA520‒4 ms, XTSA570‒4 ms, XTSA620‒5 ms, and XTSA780‒6 ms. The images were acquired as 14-bit.

### 2.3. Quantitative analysis

The count of CD31^+^ endothelial cells, αSMA^+^ smooth muscle cells, BAX^+^ apoptotic cells, and Aβ^+^ cells was quantified using the HighPlex FL module in HALO^®^ software (v.3.1.3, Indica Labs, Albuquerque, NM). Nuclei with a DAPI area greater than 9.5 μm^2^ were retained, and the cytoplasm was defined as the region extending 6 μm outward from the nuclear contour. The average fluorescence intensity within the nuclear and cytoplasmic regions was calculated, and cells with fluorescence intensity above the threshold were considered positive, while those below were considered negative. The Object Colocalization module in HALO^®^ software was used to calculate the staining area and intensity of Aβ and pTau above the fluorescence threshold. To avoid any potential influence of autofluorescence on the statistical results, the fluorescence threshold for each staining channel on each slide was manually adjusted by an experienced pathologist who was blinded to the group assignments. All data were normalized to the tissue area and were expressed as cells per unit area, staining area, or fluorescence intensity. Vascular units were defined as contiguous regions of CD31^+^ cells, and amyloid deposition within these units was quantified as a measure of cerebral amyloid angiopathy (CAA). The Proximity Analysis module in HALO^®^ software was used to quantify the number of pTau in a circle with a radius of 5 μm or a concentric circle with a width of 5 μm with the geometric center of the vascular unit as the center. The data are normalized into tissue area and displayed in terms of pTau density.

### 2.4. Statistical analysis

Remove any abnormal data from the original data that was greater than the population mean + twice the standard deviation. To ensure the robustness of outlier identification, we performed Dixon’s Q Test on all data points identified as potential outliers. The Q values for these data points were calculated and compared to the critical Q values at a 95% confidence level. All identified outliers had Q values exceeding the critical threshold, confirming their exclusion from the analysis. Group comparisons were performed using unpaired double-tailed t-tests for continuous variables and chi-square tests for categorical variables. Pearson correlation coefficients were calculated to examine the relationships between age and pathological markers. Statistical significance was set at p < 0.05. Statistical analysis and visualization were performed using GraphPad Prism (v.8.0.2) and custom R (v.4.3.2) code. All single data points used for statistical analysis are detailed in S2 and S3 Tables.

## 3. Results

### 3.1. The severity of atherosclerosis is associated with neurodegenerative changes in the human brain

**Fig 1:**
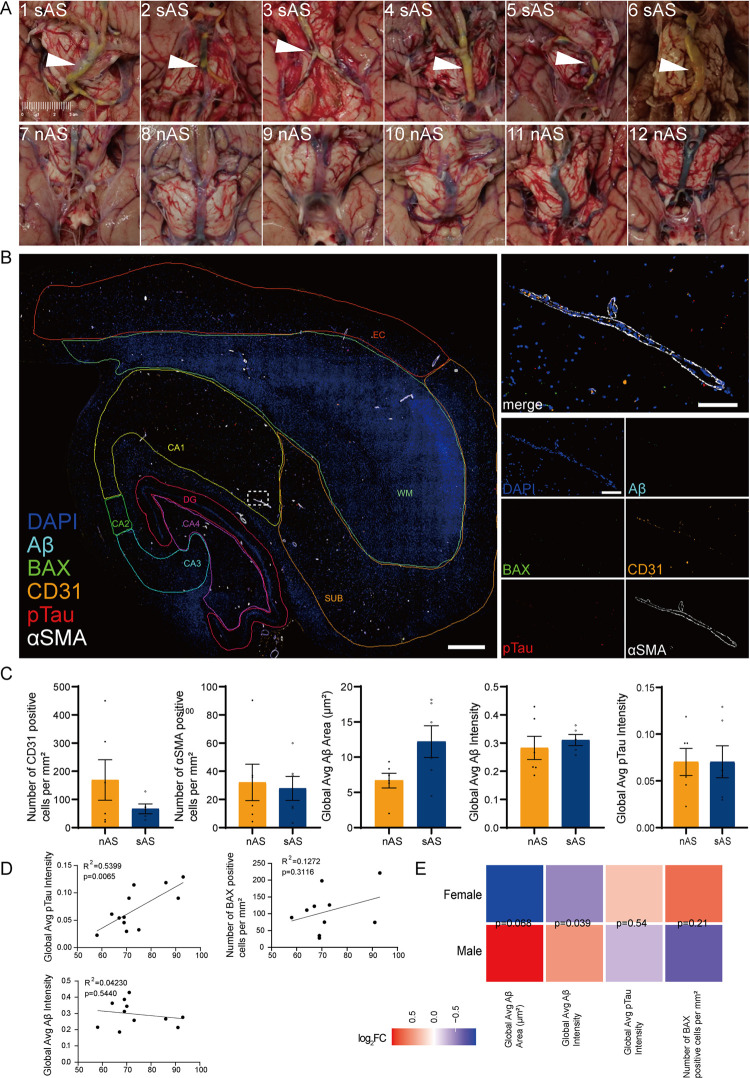
Comparison of pathological characteristics between the nAS and sAS groups. **(A)** Representative images of the circle of Willis arteries showing more severe arteriosclerosis in the sAS group (upper) than in the nAS group (lower). **(B)** Multiplex immunohistochemical staining of HP‒EC sections showing Aβ (cyan), BAX (green), CD31 (orange), pTau (red), and αSMA (white) expression and DAPI (blue). HP‒EC sections include 8 subregions: EC, SUB, CA1, CA2, CA3, CA4, DG, and WM, scale bar = 1 mm (left). High-magnification view of vascular regions, scale bar = 100 μm (right). **(C)** Quantitative analysis of the number of CD31^+^ cells per mm^2^, the number of αSMA^+^ cells per mm^2^, the Aβ area, the Aβ staining intensity, and the pTau staining intensity. The data are presented as the means ± SEMs (n = 6 per group). Unpaired t-tests were used to assess significance. **(D)** Correlation analysis of the pTau staining intensity, Aβ staining intensity, and the number of BAX-positive cells per mm^2^ with age. Linear regression models indicated significant correlations. **(E)** Heatmap comparing the fold changes of the Aβ area, the Aβ staining intensity, the pTau staining intensity, and the BAX-positive cell density between male and female groups, illustrating the log_2_FC of each parameter, calculated as the ratio of the mean values in the male group to the female group (or vice versa). Color scale represents the magnitude and direction of the log_2_FC, with red indicating higher values in males, blue indicating higher values in females. Unpaired t-tests were performed to assess the statistical significance of the differences between sexes, and the corresponding p-values are annotated within each colored block.

We analysed HP‒EC sections from individuals grouped by atherosclerosis severity (nAS group, n = 6; sAS group, n = 6) to assess vascular and neurodegenerative changes (Fig 1A). While our grouping is based on atherosclerosis severity, we note that arteriolosclerosis may also be present in the tissue sections, particularly in the sAS group. To evaluate changes in vascular structure, cell death, and neurodegeneration, we developed a multiplex immunohistochemistry panel that included vascular endothelial cell marker CD31, vascular smooth muscle cell marker αSMA, apoptotic cell marker BAX, and well-studied neurodegenerative markers including Aβ protein marker 6E10 and neurofibrillary tangles (NFTs) marker pTau, we divided the HP‒EC region into 8 subregions, including the entorhinal cortex (EC), subiculum (SUB), hippocampus (CA1, CA2, CA3 and CA4), dentate gyrus (DG), and white matter (WM) (Fig 1B). Tissue-wide quantitative analysis revealed that the vascular endothelial cell density (CD31^+^) and smooth muscle cell density (αSMA^+^) were lower in the sAS group than in the nAS group. Conversely, Aβ area and Aβ staining intensity were elevated in the sAS group, although these differences did not reach statistical significance. No changes were observed in pTau staining intensity at the tissue-wide level (Fig 1C).

Correlation analyses revealed a significant positive association between pTau staining intensity and age (R^2^ = 0.54, p = 0.0065), whereas other markers, including Aβ and BAX, did not correlate significantly with age (Fig 1D). The intensity of Aβ staining was correlated with sex (p < 0.05, Fig 1E). These findings suggest that atherosclerosis-induced vascular changes are associated with amyloid deposition and apoptosis, with age exerting a stronger influence on pTau pathology, and that sex has a greater impact on Aβ staining intensity.

### 3.2. Severe atherosclerosis is associated with increased cerebrovascular cell death

**Fig 2:**
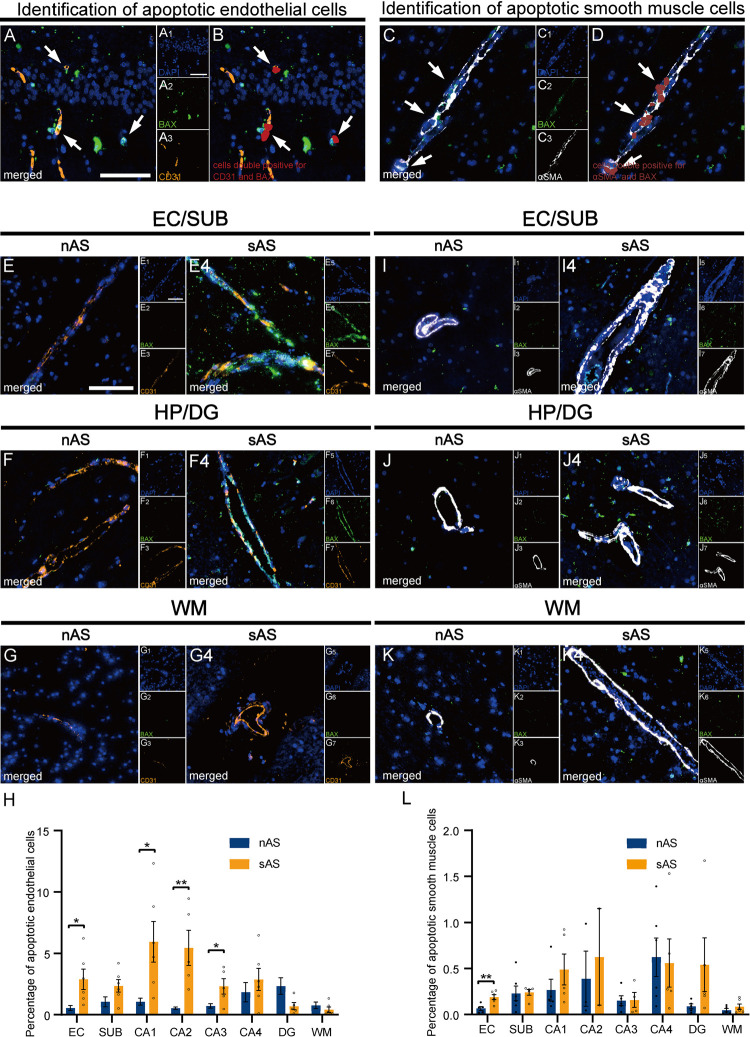
Apoptosis of cerebrovascular cells in the sAS group. **(A)** Identification of apoptotic endothelial cells (cells double positive for CD31 and BAX). **(B)** Identification of apoptotic endothelial cells via HALO^®^ software with adjusted positive thresholds for CD31 and BAX. **(C)** Identification of apoptotic smooth muscle cells (cells double positive for αSMA and BAX). **(D)** Identification of apoptotic smooth muscle cells via HALO^®^ software with adjusted positive thresholds for αSMA and BAX. **(E‒G)**. Representative images of apoptotic endothelial cells in EC, HP, and WM. **(H)** Quantitative analysis of the percentage of apoptotic endothelial cells across regions. **(I‒K)** Representative images of apoptotic smooth muscle cells in the same regions. **(L)** Quantitative analysis of the percentage of apoptotic smooth muscle cells. Scale bar = 100 μm. All data are presented as the means ± SEMs, and unpaired t-tests were performed (*p < 0.05, **p < 0.01).

To investigate the role of atherosclerosis in vascular cell apoptosis, we quantified CD31^+^BAX^+^ endothelial cells and αSMA^+^BAX^+^ smooth muscle cells in HP‒EC subregions (Fig 2A‒D). In the sAS group, the proportion of apoptotic endothelial cells was significantly elevated in HP, SUB, and EC, with the greatest increases observed in the CA2 (p < 0.01, Fig 2E‒H, S1 Fig 1A‒1B). The proportion of apoptotic smooth muscle cells was significantly increased in EC (p < 0.01, Fig 2I‒L, S1 Fig 1C‒1D), it also increased in CA1, CA2, and DG, but the differences did not reach statistical significance.

These results highlight the selective vulnerability of vascular cells in specific HP–EC subregions to atherosclerosis-related stress, suggesting a potential association between apoptosis and BBB dysfunction.

### 3.3. Severe atherosclerosis is associated with increased CAA

**Fig 3:**
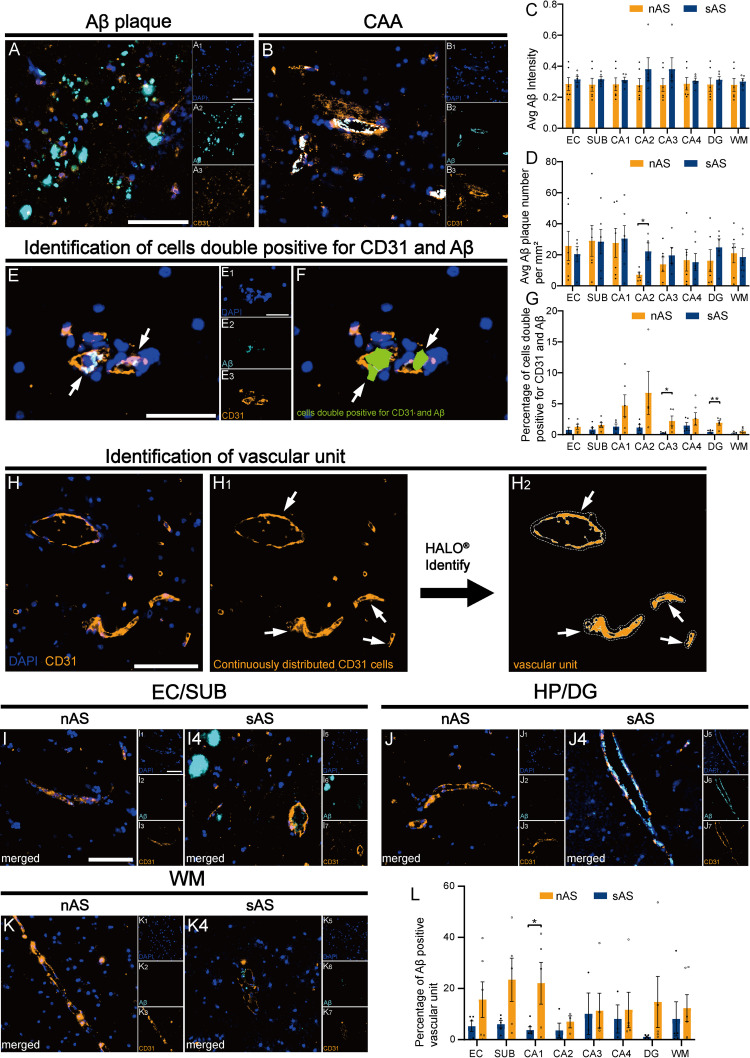
CAA in the sAS group. **(A and B)** Representative images of Aβ plaques and CAA following staining for Aβ (cyan), CD31 (orange), and DAPI (blue). **(C)** Quantitative analysis of the Aβ fluorescence intensity. **(D)** Comparison of the number of Aβ plaques per mm^2^ between the groups. **(E)** Identification of CD31^+^Aβ^+^ cells. **(F)** Identification of CD31^+^Aβ^+^ cells via HALO^®^ software with adjusted positive thresholds for CD31 and Aβ. **(G)** Quantitative analysis of the percentage of CD31^+^Aβ^+^ cells. **(H‒K)** Visualization of vascular units defined by contiguous CD31^+^ cells and their colocalization with Aβ. **(L)** Quantitative analysis of the percentage of Aβ^+^ vascular units across regions. Scale bar = 100 μm. All data are presented as the means ± SEMs, and unpaired t-tests were performed (*p < 0.05, **p < 0.01).

We next examined the relationship between atherosclerosis and amyloid deposition in the brain. Although Aβ intensity was elevated in all analyzed regions of the sAS group, no significant difference in Aβ plaque density was observed in other regions except the hippocampal CA2 region. This suggests that the increased Aβ deposition in the sAS group is not due to plaque formation but likely results from CAA, where Aβ accumulates on the walls of cerebral blood vessels (Fig 3A‒D).

The colocalization ratio of Aβ with vascular endothelial cells was significantly increased in HP–EC, particularly in DG and CA3 regions (p < 0.05, Fig 3E‒G). We defined the vascular unit as the region enclosed by continuously distributed CD31 positive endothelial cells (Fig 3H, Methods). As expected, the proportion of Aβ-positive vascular units was significantly higher in the sAS group, particularly in the hippocampal CA1 area (p < 0.05, Fig 3I‒L), suggesting that atherosclerosis selectively exacerbates vascular amyloid pathology rather than the overall plaque burden.

### 3.4. Severe atherosclerosis is associated with increased perivascular neurodegeneration

**Fig 4:**
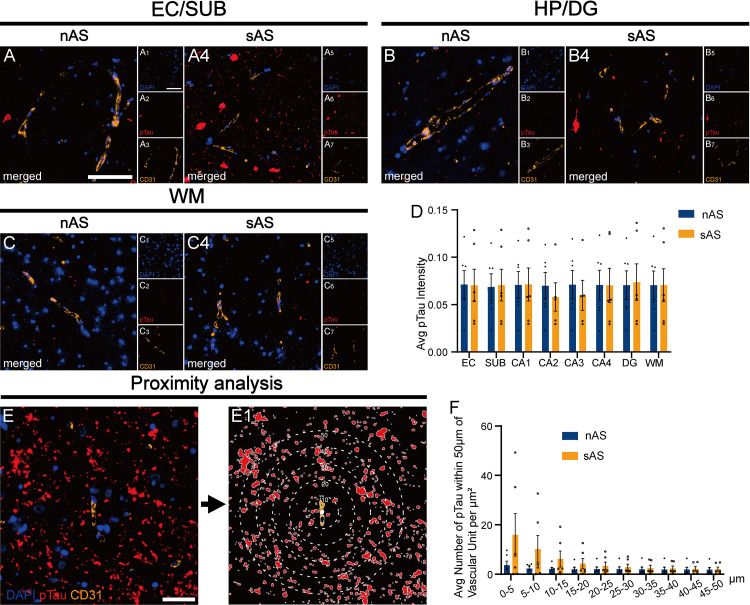
Perivascular tau pathology in the sAS group. **(A‒C)** Representative images of pTau deposition near vascular units in the EC, HP, and WM. **(D)** Quantitative analysis of the pTau fluorescence intensity. **(E)** The pattern of analysis of the pTau density within 5 μm annuli around vascular units, extending up to 50 μm. **(F)** Quantitative analysis of the number of pTau within 50 μm of vascular units per mm^2^. Scale bar = 100 μm. All data are presented as the means ± SEMs, and unpaired t-tests were performed.

To investigate whether atherosclerosis exacerbates neurodegeneration, we assessed NFTs by measuring pTau intensity and density in the brain. Although the tissue-wide pTau intensity did not differ significantly between the groups (Fig 4A‒D), the perivascular pTau density within 20 μm of vascular units was significantly greater in the sAS group than in the nAS group (Fig 4E‒F), although there was no statistical difference between the two groups at each distance range (0‒5 μm p = 0.19, 5‒10 μm p = 0.19, 10‒15 μm p = 0.24, 15‒20 μm p = 0.23). This gradient effect diminished with increasing distance from vascular units, disappearing beyond 40 μm. These findings indicate that atherosclerosis-driven vascular dysfunction promotes localized tau pathology, with implications for perivascular neurodegeneration.

## 4. Discussion

This study elucidates the relationship between severe atherosclerosis and neurodegenerative changes in HP–EC, highlighting the critical role of vascular dysfunction in AD pathology. Our findings demonstrate that severe atherosclerosis is associated with (1) increased apoptosis of vascular endothelial and smooth muscle cells, (2) elevated CAA, and (3) localized tau pathology in perivascular regions.

The significant increase in the number of apoptotic endothelial and smooth muscle cells observed in the sAS group suggests an association between atherosclerosis and disruption of cerebrovascular integrity, potentially compromising BBB function. Vascular cell apoptosis impairs the structural and functional integrity of blood vessels, facilitating the leakage of toxic molecules and inflammatory mediators into the brain parenchyma. These findings align with those of prior studies linking vascular cell death to neuroinflammation and impaired clearance of Aβ, which may be associated with neurodegenerative processes in AD [5,11,15,17].

Aβ deposition predominantly occurred near vascular units in the sAS group, underscoring the role of atherosclerosis in promoting CAA [18,19]. While the density of parenchymal Aβ plaques did not differ significantly between most groups, the increase in vascular Aβ deposition highlights the impact of impaired Aβ clearance. These findings complement studies suggesting that vascular amyloid deposition may be associated with atherosclerosis‒induced alterations in cerebral blood flow and BBB permeability. Given the role of CAA as an independent risk factor for cognitive decline, these results emphasize the importance of addressing vascular health to prevent AD.

Localized tau accumulation near vascular units in the sAS group reveals a potential mechanism suggesting an association between atherosclerosis and tau pathology. The observed perivascular gradient of pTau staining intensity suggests that vascular dysfunction may contribute to the development of a microenvironment conducive to tau aggregation, possibly mediated by localized inflammation and reduced protein clearance [20–22]. This finding aligns with emerging evidence that vascular insults may be associated with the progression of tau pathology and neuronal damage, contributing to cognitive impairment in AD.

These results support the growing recognition that vascular and neurodegenerative pathologies in AD are interdependent rather than isolated processes [23,24]. Our study extends prior findings by demonstrating that severe atherosclerosis is associated with specific vascular and neurodegenerative changes in HP–EC, a region critical for learning and memory. The selective vulnerability of HP–EC subregions to vascular cell apoptosis and amyloid deposition highlights the complex interplay between vascular health and neuronal integrity.

This study has several limitations. The relatively small sample size may limit the generalizability of the findings, and the fact that the samples were postmortem samples precluded direct assessment of dynamic processes such as protein clearance. Additionally, while we identified correlations between atherosclerosis severity and the levels of pathological markers, the causative mechanisms remain to be fully elucidated. It should also be noted that the majority of sAS group patients died from cardiac failure, which could potentially confound our observations of vascular apoptosis. Systemic hypoperfusion or hypoxia associated with terminal cardiac events might exacerbate cerebrovascular damage independent of atherosclerosis severity. Future studies involving longitudinal clinical cohorts and animal models could provide deeper insights into the temporal and mechanistic relationships between vascular dysfunction and neurodegeneration.

In conclusion, this study highlights the pivotal role of severe atherosclerosis in driving cerebrovascular and neurodegenerative changes in HP–EC, emphasizing the interconnectedness of vascular and neuronal health in AD. By identifying associations between vascular cell apoptosis, CAA, and perivascular tau pathology, our findings provide novel insights into the mechanisms underlying neurodegeneration and underscore the importance of targeting vascular health to mitigate AD progression.

## Supporting information

S1 Fig Multiple microscopic field stitched images of apoptotic vascular cells. (A‒B) Representative stitched images of apoptotic endothelial cells in HP in the nAS group (A) and the sAS group (B). (C‒D) Representative stitched images of apoptotic smooth muscle cells in EC in the nAS group (C) and the sAS group (D). Scale bar = 100μm.
(TIF)

S1 Table Demographics of the subjects. (XLSX)

S2 Table All the single data points used for the unpaired two-tailed t-test. (XLSX)

S3 Table All single data points used for Pearson correlation analysis. (XLSX)
