# Supplementary figures and images for: Atherosclerosis is associated with amyloid and tau pathology via blood–brain barrier dysfunction in the hippocampus of aged human brains
Source: PLoS One. 2025 Jun 11;20(6):e0324652. doi: 10.1371/journal.pone.0324652 (PMC12157335; doi:10.1371/journal.pone.0324652)

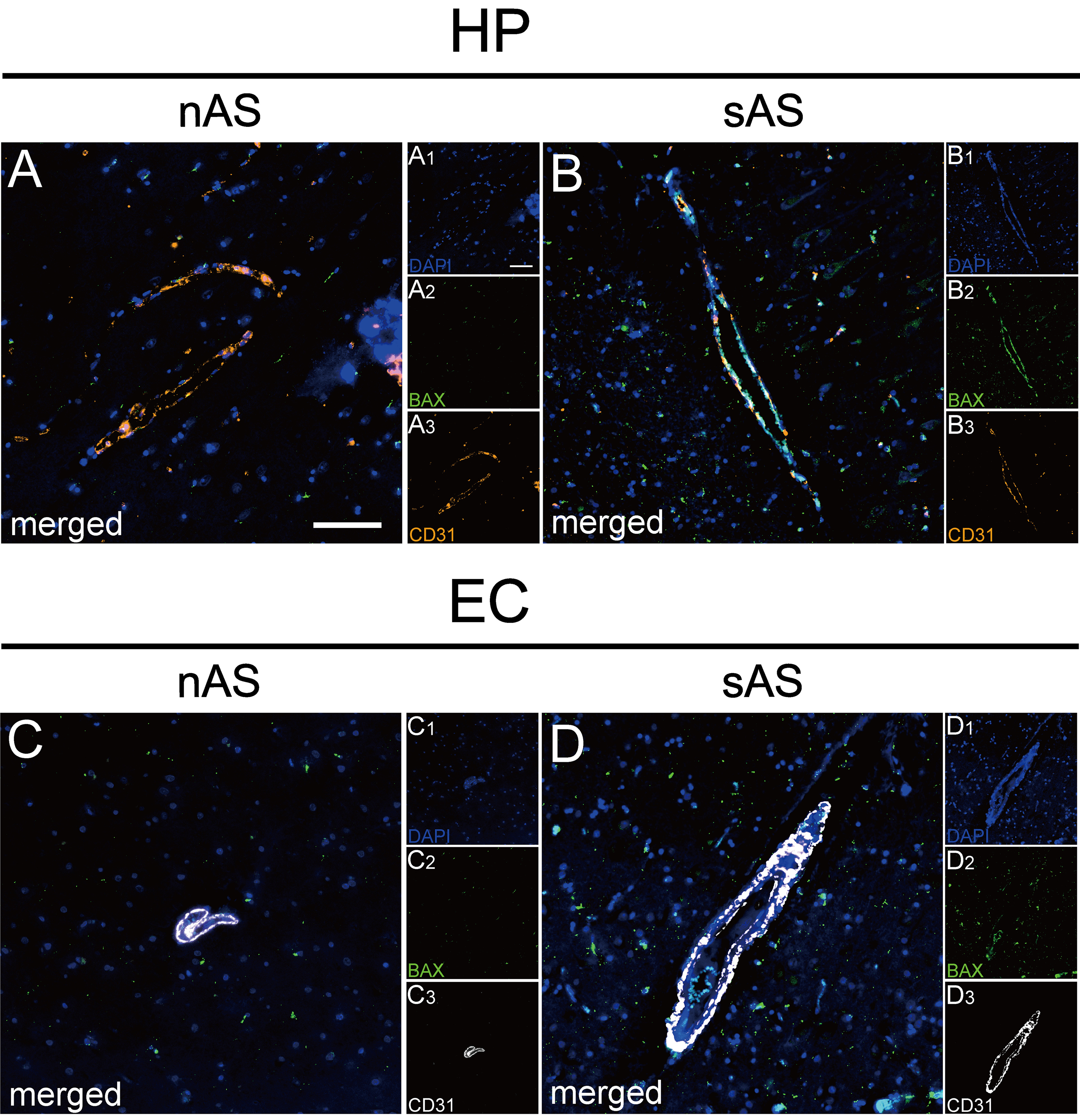

Supplement: S1 Fig — (A‒B) Representative stitched images of apoptotic endothelial cells in HP in the nAS group (A) and the sAS group (B). (C‒D) Representative stitched images of apoptotic smooth muscle cells in EC in the nAS group (C) and the sAS group (D). Scale bar = 100μm. (TIF) [file pone.0324652.s001.tif]
